# Effect of Deposition Pressure and Temperature on Tungsten Thin-Film Heater for Phase-Change Switch Applications

**DOI:** 10.3390/mi15050576

**Published:** 2024-04-26

**Authors:** Sheng Qu, Jihua Zhang, Libin Gao, Hongwei Chen, Yao Ding

**Affiliations:** 1 School of Integrated Circuit Science and Engineering, University of Electronic Science and Technology of China, Chengdu 610054, China; qushengqs@126.com (S.Q.); hwchen@uestc.edu.cn (H.C.); dyao9916@163.com (Y.D.); 2 State Key Laboratory of Electronic Thin Films and Integrated Devices, University of Electronic Science and Technology of China, Chengdu 610054, China

**Keywords:** microheater, tungsten film, magnetron sputtering, phase-change switch

## Abstract

Tungsten (W) film is increasingly utilized in various microheater applications due to its numerous advantages. These advantages include a high melting point, positive constant temperature coefficient of resistance (TCR), good mechanical stability, and compatibility with semiconductor processes. In this paper, deposition parameters for enhancing the properties of W film were investigated, and an optimized microheater was fabricated. It was found that the deposition temperature and pressure can modify the TCR to be negative or positive and the crystalline phase of W films to be alpha phases or mixed with beta phases. A W film deposited under 650 °C with a pressure of 1 pa has a positive TCR and pure alpha phase crystalline structure. We applied this optimized W film as a microheater in an RF phase-change switch (RFPCS), and the maximum voltage of the optimized W microheater increased by at least 48% in this work. By optimizing the microheater, the phase-change switch can be successfully actuated in both on and off states, demonstrated by the Raman results of the phase-change material. A voltage pulse of 20 V/200 ns was enough to turn the switch off with MΩ, and 11 V/3 μs could turn the switch on with 138 Ω. The optimized microheater and device can cycle 500 times without failure. The insertion loss and isolation of the device at 20 GHz was 1.0 dB and 22 dB.

## 1. Introduction

Microheaters serve as pivotal thermal control devices in the realm of microelectronics, facilitating precise temperature control and regulation and finding extensive application across various domains including sensors [1,2,3], microfluidic channels [4,5,6], calorimetry [7,8,9], and phase-change devices such as a phase-change memory [10,11,12] and phase-change switch (PCS) [13,14,15,16,17]. In phase-change device applications, phase-change materials are capable of transitioning between two distinct states: an amorphous state with high resistance and a crystalline state with low resistance. An intense pulse for a short time causes the material to rapidly elevate its temperature beyond the melting point (T_m_), transitioning it into a liquid state. Subsequent rapid cooling causes the atoms to solidify in a disordered arrangement, completing the shift from the crystalline to the amorphous state. Meanwhile, the amorphous-to-crystalline transition requires a pulse that keeps the temperature of the PCM below the melting point but above the crystallization temperature (T_c_) for a period of time, allowing the atoms to reassemble into an organized arrangement. Therefore, thermal control of microheaters is crucial to the functionality of phase-change materials. With the recent ongoing advancement of RF phase-change switch (RFPCS) technology, expectations concerning the design and performance of microheaters for applications in indirectly heated structures have been notably elevated, for which an additional microheater is required to supply heat to phase-change materials, such as GeTe [18].

Tungsten (W) thin film boasts outstanding properties with a high melting point (3380 °C), exceptional thermal conductivity, positive constant temperature coefficient of resistance (TCR), and chemical stability [19], rendering it an optimal choice as a microheater in an RFPCS. However, the majority of existing work on RFPCSs predominantly delves into enhancing the performance of phase-change films and devices, with relatively few reports having been devoted to a detailed study of W films for phase-change switch applications [20,21,22,23]. For example, how to ascertain the applicability of a W film as a microheater for enabling the functionality of phase-change switches, as well as how to optimize the deposition parameters of magnetron sputtering to achieve enhanced stability of a W film as a microheater have not been analyzed. Focusing on these questions, the deposition parameters of W films are investigated to mitigate damage encountered during phase-change switch measurements, and a W microheater with enhanced stability is fabricated within an indirectly heated RFPCS application.

**Figure 1:**
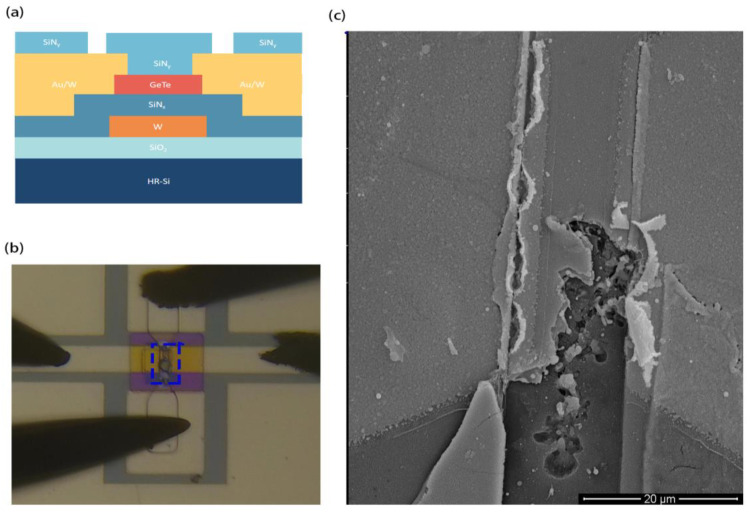
(**a**) Cross-section representative schematic of GeTe-based indirectly heated phase-change switch fabrication. (**b**) Optical graph of phase-change switch during application of pulse voltage when damage occurs. (**c**) SEM image of fabricated device of blue dashed box in (**b**).

Figure 1a shows a schematic of the cross-section structure in a GeTe-based indirectly heated RFPCS. The process steps of fabricating are described in [24]. The phase-change switch was fabricated in several sequential steps. Beginning with a 650 μm thick single-side polished silicon (p-type) substrate, 100 nm thermally grown SiO_2_ thin film was applied as the substrate insulator. Patterning was carried out using the lift-off technique, followed by deposition of a 100 nm layer of W film to serve as the microheater for triggering the phase-change layer. Next, a 50 nm SiN_x_ film was deposited using Plasma-Enhanced Chemical Vapor Deposition (PECVD) and patterned by the Inductive Coupled Plasma (ICP) dry-etching method. Subsequently, a 150 nm thick GeTe film was deposited as the phase-change layer via RF sputtering and then patterned using the lift-off technique. Following this, a 180 nm thick W/Au metal film was deposited and subsequently patterned using lift-off techniques to serve as the RF electrode layer. To protect the GeTe film from excessive exposure to air, a 120 nm SiN_y_ dielectric passivation film was deposited by PECVD. Openings were also dry-etched to create electrical pads for switch actuation.

Figure 1b shows an optical microscope view of the damage that occurred during the switching test, where four probes were used to apply a pulsed voltage and measure the device’s resistance. Figure 1c shows the SEM image in the blue dashed box of Figure 1b. To turn the switch off, a voltage pulse was applied to the W microheater (the upper and lower probes in Figure 1b). The pulse width was kept at 200 ns, and the amplitude of the pulse was increased by steps of 0.5 V. Damage occurs when the amplitude reaches 25 V and the switch fails to achieve the off state. The W film in the center region showed significant holes and cracks, which is not desired during testing. The resistance of the microheater as a function of temperature is important for device stability. It is known from a previous report [19] that a W film has a positive and constant TCR under a certain range of temperatures. However, it was found in this paper that the TCR varies with deposition conditions and is associated with damage to the device. In order to investigate the reason for this damage, the influence of the deposition conditions of W films was investigated, and the relationship between the TCR of W films and deposition parameters was also studied.

## 2. Experimental Procedure

Tungsten films with a thickness of 100 nm were prepared for characterization on p-type (100) silicon substrates with a resistivity of 10^3^ Ω·cm and a 100 nm oxide layer. The substrates were degreased in acetone and deionized (DI) water with ultrasonication for 5 min and then blown dry with nitrogen (N_2_). There were four samples, marked as A, B, C, and D. All the samples were deposited using a tungsten target of 99.99% purity by DC magnetron sputtering with an Ar flow of 35 sccm. The tungsten target was a round target with a 3 inch diameter and 3 mm thickness. The distance from the target to the substrate was 20 cm, and the target was inclined with a 40° angle in a cylindrical chamber with a diameter of 45 cm. Considering the resistivity and adhesion of the film, a sputtering power of 200 W and sputtering pressure of 0.3 pa at room temperature were used as the initial conditions to deposit W films. Sample A and C were deposited at room temperature (RT) with a pressure of 0.3 Pa and sputtering power of 200 W. Sample B and D were deposited at 650 °C with a pressure of 1 Pa and sputtering power of 100 W. The sputtering parameters are listed in Table 1. The film structures were characterized using X-ray diffraction (XRD) with CuKα radiation (k = 0.1540 nm) employing the Bruker D8 Advance instrument. Scanning Electron Microscopy (SEM) was carried out using the Inspect F microscope from FEI Company. The resistance–temperature (R-T) curve diagram was measured using the Quantum Design- PPMS 9 instrument with a heating rate of 10 °C /min from RT to 350 °C. For the testing of the W microheater’s electrical properties, the voltage pulse was triggered by an Agilent 8114A high-power pulse generator, and the resistance measurement was detected by a Keithley 2400 digital source meter. Agilent 8114A was set as the burst mode for single-trigger and continuous modes for actuating the switch. The Keithley 2400 Digital Source Meter is capable of detecting voltage, current, and resistance, while also functioning as a voltage and current source. The surface morphology was obtained by a 3D Surface Profiler vk-x3000 of KEYENCE, which has the ability to measure a wide range of samples (from 1 nm to 50 mm) and nano-/micro-/millimeter measurements can be accomplished using this one system. For Scanning Transmission Electron Microscope (STEM) imaging, rough cross-sections were initially extracted from the sample surface through Focused Ion Beam (FIB) lift-out techniques and subsequently affixed onto a copper TEM grid using FIB-deposited platinum. The FIB instrument was using Helios 5CX and TEM was performed using a Tecnai G2 F20 S-TWIN microscope from FEI Company, Hillsboro, OR, USA. The Energy-Dispersive Spectrometer (EDS) scan was conducted using equipment from Oxford Instruments. RF measurements were performed using Ground–Signal–Ground (GSG) probes from GGB industries with a 150 μm pitch, and the Vector Network Analyzer (VNA) was Agilent N5242 PNA-X and was used to measure S-parameters of the switch between the on- and off state from 10 MHz to 20 GHz.

## 3. Results and Discussion

**Figure 2:**
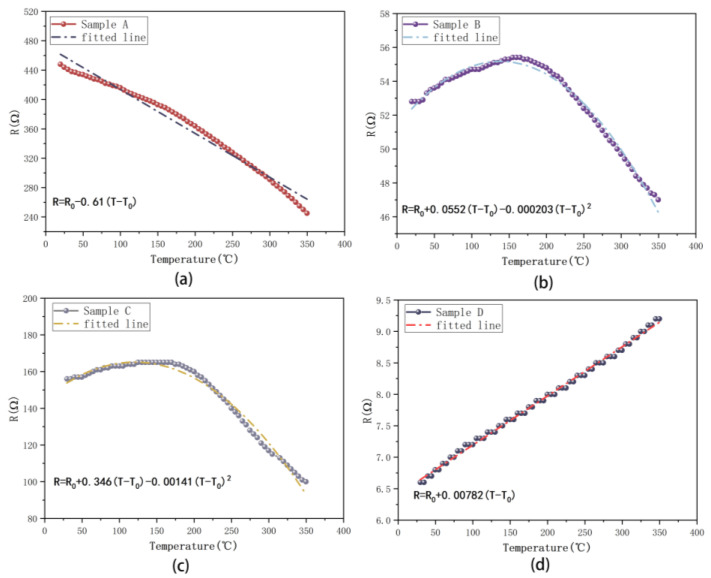
R-T curves and fitted lines of (**a**) sample A; (**b**) sample B; (**c**) sample C; (**d**) sample D.

Figure 2 shows the resistance–temperature (R-T) curves and fitted lines of four samples. It can be observed from Figure 2a,b that a higher deposition temperature results in lower resistance. For instance, the resistance of sample A is 450 Ω at RT, while that of sample B is 53 Ω at RT, with the latter being deposited at 650 °C. Although the resistance of sample C decreases to 154 Ω at RT compared to sample A, it is still not as low as that of sample B. It is worth noting that there is an increase in resistance within the range of room temperature up to 200 °C, while the resistance begins to decrease from 200 °C to 350 °C in both sample B and sample C. The fitted equations of the lines are shown in each sub-figure of Figure 2. As the temperature rises from RT to 350 °C, the resistance of sample D increases from 6.6 Ω to 9.2 Ω. It is notable that sample D is the only sample whose resistance at 350 °C is higher than the initial resistance R_0_ (resistance at RT) and that it has the lowest resistance among these samples. A preliminary conclusion can be drawn from the R-T curves, which is that the resistance of W films deposited at 650 °C is lower compared to those deposited at room temperature, and that a significant decrease in the resistance of W film occurs under a pressure of 1 Pa and temperature of 650 °C. The resistance of a W film deposited at RT and 0.3 Pa exhibits a linear relationship of a fitted line with the resistance, decreasing as shown in Figure 2a. Figure 2d also shows a linear relationship but with the resistance increasing. However, as the deposition pressure or temperature increases, this relationship becomes nonlinear, as shown in Figure 2b,c.

**Figure 3:**
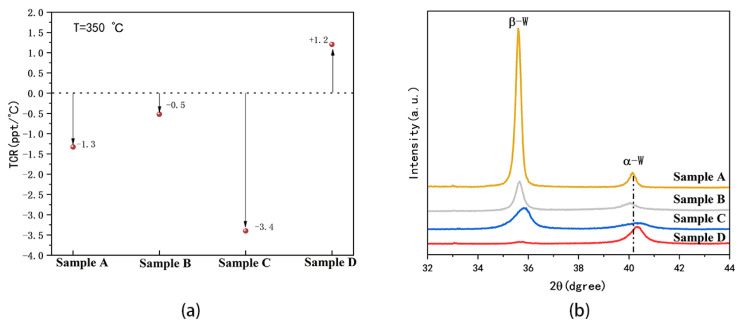
(**a**) TCRs of four samples at T = 350 °C. (**b**) XRD patterns of four samples.

Figure 3a depicts the TCRs of the four samples. The resistance of the film can be obtained from the linear model:

R = R_0_ + α_w_ (T − T_0_) R_0_

By calculating the slope of the fitted line or the first-order derivative of the equation in Figure 2, it can be concluded that the value α_w_ at 350 °C for samples A, B, C, and D is −1.3, −0.5, −3.4, and +1.2 ppt (part per thousand)/°C, respectively. When the voltage is applied to the W film, the generated current causes the Joule effect, leading to an increase in temperature, which is how the microheater works. However, a negative TCR means that the resistance decreases as the temperature rises, with the applied pulse voltage remaining constant, and the current becomes progressively higher, which in turn generates more Joule heat. Although this situation favors a rapidly rising temperature within certain limits, the negative TCR is likely to contribute to film cracking and device damage, as shown in Figure 1.

It is well known that tungsten is classified into two structures: alpha-W (α-W) with a stable body-centered cubic structure [25] and beta-W (β-W) with a cubic A15 metastable structure [26,27]. At room temperature, the bulk resistivity of β-W is approximately 5–10 times higher than that of α-W [28]. Figure 3b shows XRD patterns investigating the crystalline structure of four samples. The diffraction patterns of samples A, B, and C exhibited α-W mixed with β-W phase structure for the peaks of 2θ at 35.6° and 40.2°, corresponding to (200) of β-W and (110) of α-W, respectively [29]. Sample D consists solely of the α-W phase, characterized by the highest (110) diffraction peak intensity. Therefore, combining the XRD result with the R-T curves, a film with only an α-W structure does, indeed, have a lower resistance, with sample D being an α-W structure in this work. In addition, the peaks of samples A and B are noticeably offset from those of samples C and D to a certain extent. This shift is attributed to tensile stress, induced by variations in the sputtering pressure, which are related to the existence of larger atomic plane spacing, which can be alleviated through film annealing [29].

**Figure 4:**
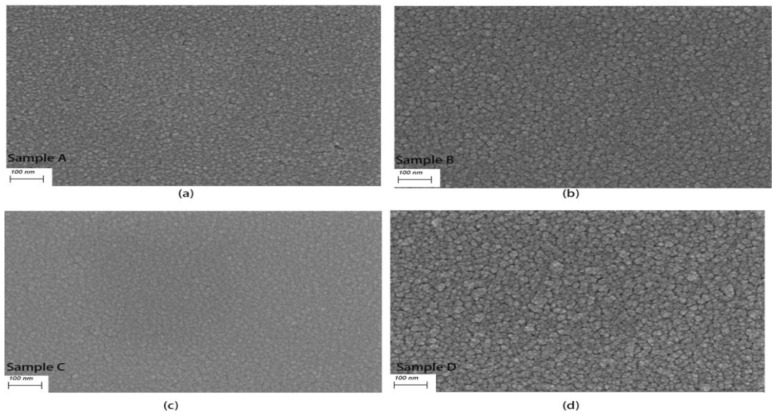
SEM images of (**a**) sample A; (**b**) sample B; (**c**) sample C; (**d**) sample D.

Figure 4 presents SEM images illustrating the four samples. It is evident that the grain sizes of samples deposited at 650 °C (Figure 4b,d) are notably larger than those deposited at room temperature (Figure 4a,c). Samples deposited at a low pressure exhibit minimal changes in grain size, as seen in Figure 4a,c. The films deposited at room temperature appear denser with increased scattering at the grain boundaries, leading to a higher resistance [30], which is consistent with the high resistivity of β-W. Smaller grains in materials typically lead to more grain boundaries, where stresses are propagated and retained. Consequently, materials with smaller grains may exhibit higher stress concentrations at grain boundaries under stress conditions. The crystalline structure of materials with small grains can be influenced by elastic distortion caused by grain boundaries, resulting in localized stress increases. This phenomenon may lead to grain deformation and distortion, thereby affecting the mechanical properties and stability of the material [31]. Therefore, sample A and sample C, characterized by smaller grains and more crystalline boundaries, exhibit higher resistance and stress. Significantly, during the measurement of the phase-change switch depicted in Figure 1, it seems that the W microheater fabricated under the deposition conditions of sample A ultimately caused damage to the device due to the excessive stress induced by Joule heating.

In order to evaluate the stress changes in the films, the grain sizes, as well as the microstress strain, were calculated based on the XRD results in Figure 3b using the Debye–Scherrer formula. The calculation results are shown in Table 2, where D = kλ/(βcos(θ)), ε = β/4tan(θ), k is Scherrer’s constant of 0.89, λ is a wavelength of 0.154 nm for Cu Kα radiation, and β is the Full Width at Half Maximum (FWHM). From Table 2, it can be seen that sample D has the largest grain size and the smallest strain, which is in accordance with the SEM results, and therefore, it can be predicted that the stress of sample D is the smallest among the four samples.

**Figure 5:**
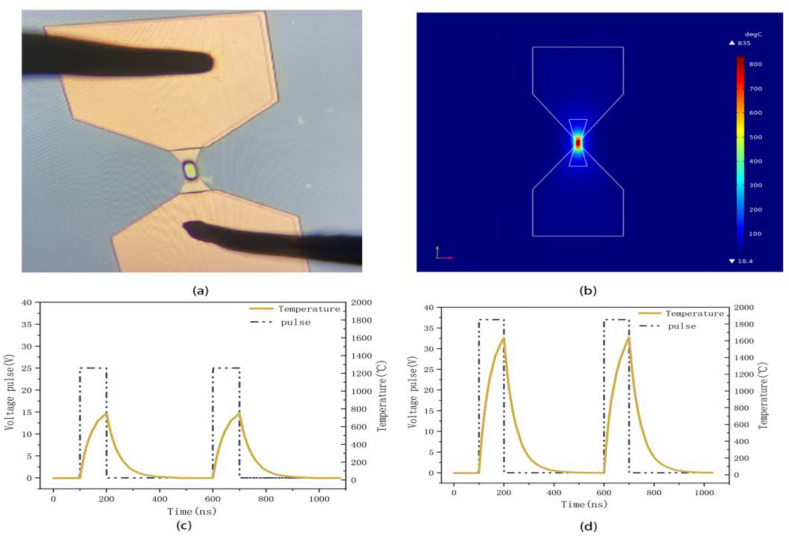
(**a**) Optical microscope graph of W microheater after applying voltage pulse with 25 V/200 ns. (**b**) Simulation with temperature distribution of W microheater by FEM method. (**c**) Simulation results of temperature in central point of microheater under pulse of 25 V/200 ns; (**d**) simulation results of temperature in central point of microheater under pulse of 37 V/200 ns.

Recognizing the possibility that the film cracking observed during testing could be attributed to inappropriate deposition conditions, an optimized heater was fabricated using the deposition process of sample D and patterned using lithography technology to verify its stability under voltage pulse. The Finite Element Modeler (FEM) method was employed to simulate its temperature distribution, as illustrated in Figure 5a,b. The heat distribution was simulated by Comsol Multiphysics, coupled with the electrical circuit and solid heat physics fields. The pulse was set by the mathematical field. The related parameters are listed in Table 3. In Figure 5a, the initial resistance of the heater is 35 Ω. When subjected to multiple pulses of 25 V/200 ns, the resistance of the microheater remains at 35–40 Ω with no significant changes, indicating an improved performance compared to Figure 1. As the pulse amplitude increased more, a hot spot emerged at the central area of the microheater, which is consistent with the simulation results depicted in Figure 5b, indicating that heat predominantly concentrated in the central region of the heater. Figure 5c shows the simulation temperature of the central point, which reached as high as 750 °C at 25 V/200 ns. This temperature is sufficient to induce rapid melting and quenching of GeTe, facilitating its transition from a crystalline to an amorphous state [24], acting as an off state of phase-change switch. When the center of the heater surpasses half of its melting point temperature, prolonged usage may compromise the stability of the device [32]. Simulations provided results up to a maximum temperature close to half of the melting point of W (≈1690 °C), as shown in Figure 5d. It is evident that the optimized heater can endure pulses up to 37 V/200 ns without compromising its stability. When a 37 V/200 ns voltage pulse is applied to the microheater of Figure 5a, the resistance increases to 59 Ω, indicating that 37 V/200 ns is not yet the maximum voltage for the heater, and the microheater is still functional. Therefore, the maximum voltage of the optimized heater has increased by at least 48% compared to the results in Figure 1. The result only considered the distribution of heat, and effects between layers and interfaces should be considered in future work.

**Figure 6:**
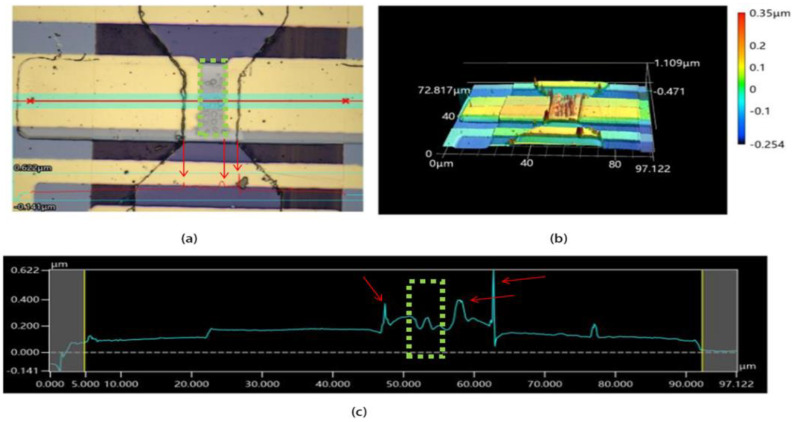
(**a**) Main image of vk-x3000 graph of phase-change switch with microheater, including process of sample A, after applying single voltage pulse with 25 V/200 ns; (**b**) 3D image of (**a**); (**c**) measured cross-section height curves as red line in (**a**).

To assess the performance of the optimized heater for an indirectly heated RFPCS, a corresponding phase-change switch was re-fabricated following the steps outlined in Figure 1a, and then compared with the phase-change switch depicted in Figure 1b. The surface morphology of the switch used in Figure 1 was analyzed using a vk-x3000 3D surface profiler, as presented in Figure 6. The main image of the device is displayed in Figure 6a, with the green dashed box indicating the central region where heat generation might be most concentrated based on the results of the simulation. Figure 6b presents a 3D surface image of Figure 6a, revealing numerous prominent spikes and bulges across the device, which demonstrates considerable surface roughness. The corresponding relative height profile was obtained by scanning the red line in Figure 6a, depicted in Figure 6c. However, contrary to expectations, the spikes and bulges at the central region of the device are not as prominent. Conversely, the spikes and bulges at the edge of the heater or the RF electrode reach heights of 200–400 nm, even exceeding the overall thickness of the device. Thermal stresses generated by the heater tend to accumulate primarily at the edges of the film, as evidenced by the red arrows in Figure 6c. When a 25 V/200 ns pulse is applied to the device, the central region of the heater experiences rapid heating, while the temperature at the edges rises gradually, creating a temperature gradient. This gradient can induce thermal stresses, resulting in the formation of spikes and bulges, ultimately leading to film cracking and device damage [33]. In addition, spikes on the edges of the device may also be induced by the process of fabrication, as lift-off processing can easily generate surface topology at the edge of features.

**Figure 7:**
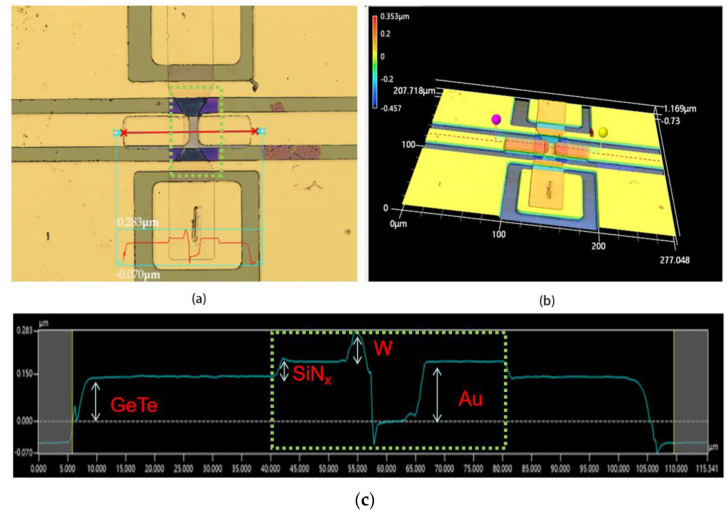
(**a**) Main image of vk-x3000 graph of phase-change switch with microheater, including process of sample D, before applying voltage pulse; (**b**) 3D image of (**a**); (**c**) measured relative height curves as red line in (**a**).

**Figure 8:**
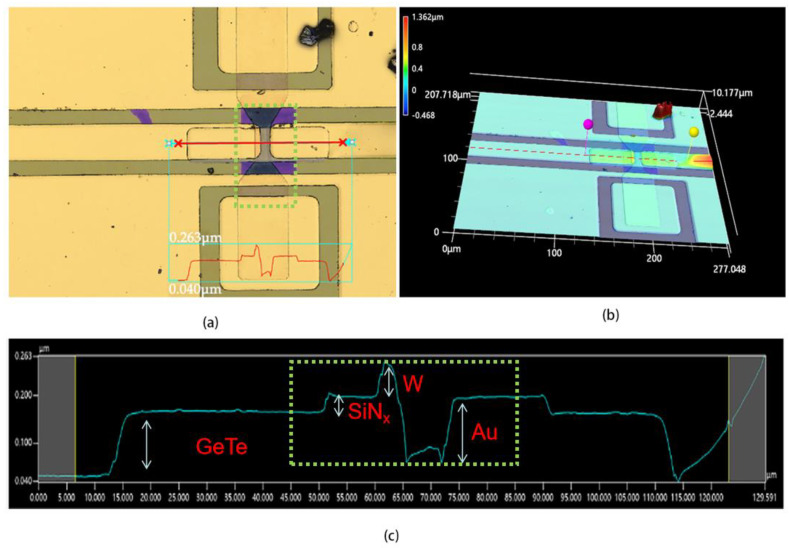
(**a**) Main image of vk-x3000 graph of phase-change switch with microheater, including process of sample D, after applying 500 pulses of 25 V/200 ns; (**b**) 3D image of (**a**); (**c**) measured relative height curves as red line in (**a**).

The devices in Figure 7 and Figure 8 are the optimized device fabricated with the microheater by a deposition process of sample D from one wafer. Figure 7 shows the optimized device before pulses were applied, and Figure 8 shows the result after applying 500 pulses with a vk-x3000 3D surface profiler. Before applying pulses, the device exhibited barely significant spikes or bulges, as shown in Figure 7a,b. It is worth noting that the curve in the dashed box region of Figure 7c fluctuates less and remains relatively flat compared to Figure 6c. However, after 500 pulses of 25 V, as shown in Figure 8c, the curve in the center region exhibits a fluctuation, with a preliminary estimate of more than 10 nm, as shown in blue arrows, suggesting that a volume expansion occurs in the center of the heater after applying 500 pulses. It is not possible to determine for the time being whether this expansion is due to the GeTe or the W thin film, since the GeTe phase transition also causes a change in volume. In addition, from Figure 7c and Figure 8c, it can be seen that the thicknesses of the GeTe, Au, SiN, and W films are about 150, 50, 80 and 180 nm, respectively.

**Figure 9:**
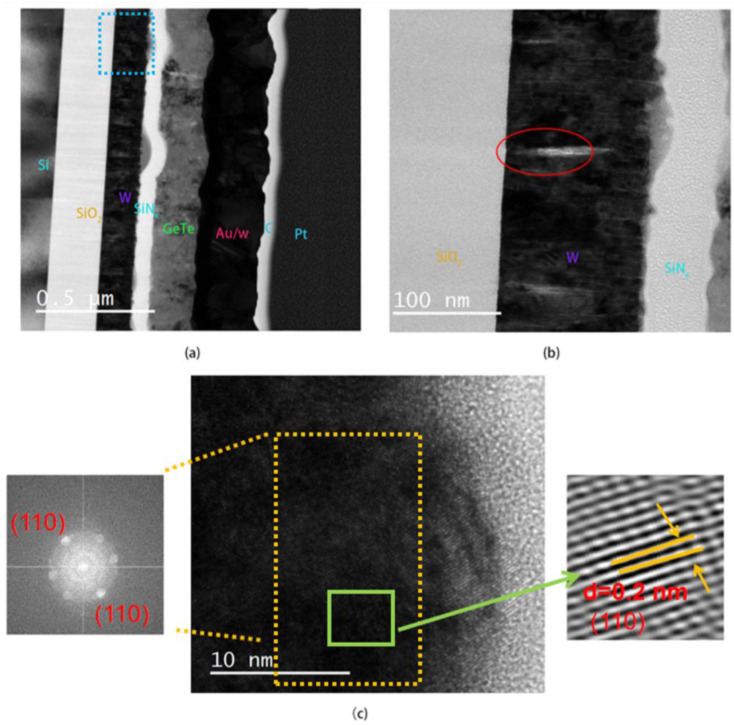
(**a**) STEM image of optimized device with pulse remaining in on state; (**b**) enlarged view of dashed box in (**a**); (**c**) high-resolution image of interface between W and SiN_x_ and associated Fast Fourier Transform (FFT) in the left sub-figure with lattice spacing calibration as shown in the right sub-figure of green box.

The cross-sectional view of the device can be observed using the FIB-TEM technique. Figure 9a shows the STEM image after FIB sampling, with Pt and C serving as protective layers. It can be seen that the area in the blue dashed box exhibits some bulging of SiN_x_. Figure 9b is the enlarged view of the dashed box region. The white bars in the red circles are voids in the film, which may be attributed to grain growth or be a result of strain from the thermal expansion after applying pulses. It can be seen in Figure 9b that the W heater is uniformly smooth overall and does not manifest obvious thermal expansion. Meanwhile the top layer of SiN_x_ appears to swell and bulge. Figure 9c displays a high-resolution image of the interface between W and SiN_x_ and the associated Fast Fourier Transform (FFT) with lattice space calibration. The diffraction ring on the left of Figure 9c is the FFT result for the orange dashed box and is attributed to α-W [28]. Through Inverse Fast Fourier Transform (IFFT) of the diffraction points in the FFT result, the image of the lattice arrangement can be obtained. As shown to the right of Figure 9c, the lattice spacing is calculated as 0.2 nm, which corresponds to the lattice spacing of (110) in the α-W (#PDF 01-1203), and this result also corresponds to the (110) peak of sample D at 40° in Figure 3b.

**Figure 10:**
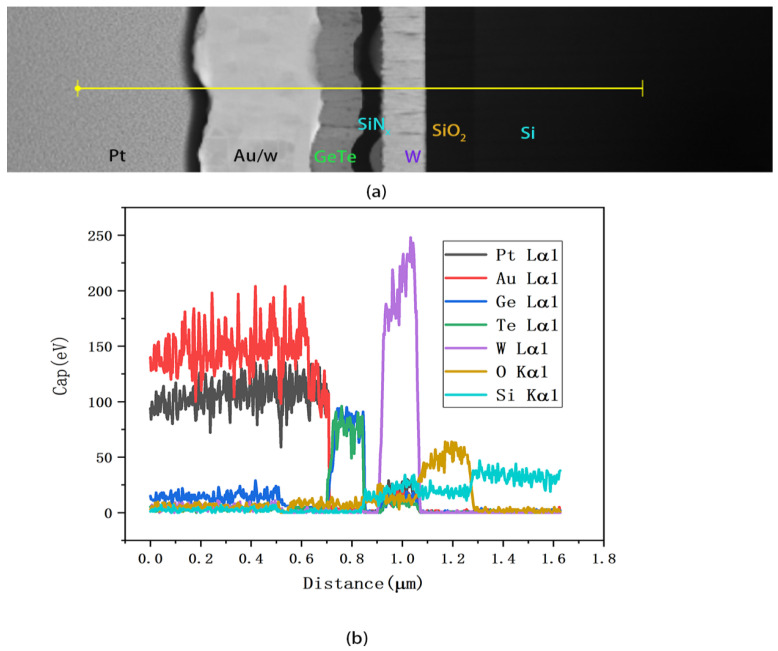
(**a**) STEM-EDS line scan of the optimized device. (**b**) EDS line scan result of (**a**).

To investigate the material degradation around the W film and the oxidation of the W film during device operation, a STEM-EDS line scan analysis was conducted, as depicted in Figure 10. The scan results in Figure 10b indicate that the oxygen element distribution is predominantly near the substrate, corresponding to the insulating SiO_2_ layer on the Si substrate. There was no significant diffusion observed between the W, Ge, and Te layers. Notably, due to the overlapping energies of the Pt Lα and Au Lα from the protective Pt layer deposition in FIB, the Pt and Au contents are located in approximately the same region in the figure. In addition, there is a partial peak overlapping of energy spectra in W Lα and Si Kα. There seems to be some diffusion of O or oxidation in W, while the content of O is extremely low in the W region based on the EDS result, and it can be determined that the oxidation of W is slight. However, it can be inferred that with repeated heating cycles, there may be a tendency for O to increase in the W region, potentially affecting the quality of W over a larger number of heating cycles. Since the number of cycles in this experiment was limited (500 times), further investigations with more heating cycles are warranted to understand the effect of the O content on the W microheater.

**Figure 11:**
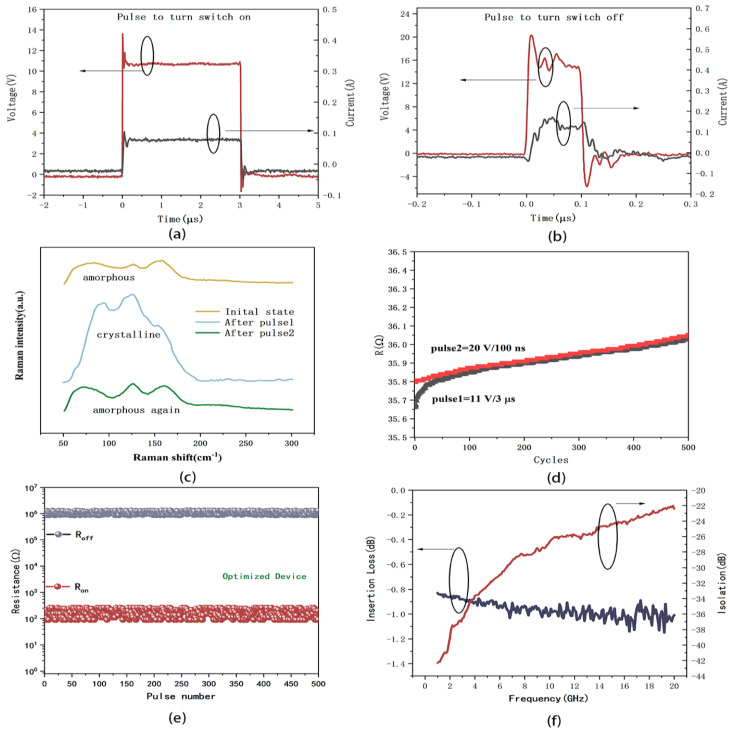
(**a**) Voltage pulse for turning switch on and corresponding current value; (**b**) voltage pulse for turning switch off and corresponding current value; (**c**) Raman spectrum of GeTe film in device before and after pulse; (**d**) endurance of W microheater for 500 cycles under pulses; (**e**) endurance of phase-change switch for 500 cycles as on- and off state; (**f**) measured RF insertion loss and isolation of fabricated device with optimized microheater.

This study proposes a method to optimize the microheater by tuning the value of TCR with a deposition parameter, enhancing the switch’s reliability. Figure 11a,b display the necessary pulse parameters of voltage and corresponding current values to switch the device on and off. Notably, it was discovered that a pulse of 25 V/200 ns was unnecessary to turn off the switch; instead, a pulse of 20 V/200 ns was sufficient. Figure 11a illustrates that when the pulse duration is long, the waveforms of pulse current and pulse voltage remain consistent. Conversely, with shorter pulse durations, the voltage amplitude becomes unstable due to induced disturbances in the equipment and parasitic parameters in the device, leading to fluctuating current values. However, it is noteworthy that the current decreases gradually, corresponding to the optimized positive resistance coefficient of the W heater.

A Raman spectrum was utilized to confirm the crystallization of GeTe following the application of pulses to the heater. The results in Figure 11c illustrate the initial amorphous state of deposited GeTe, transitioning to a crystalline state after applying 11 V/3 μs (pulse 1). The resistance of the device varied from M Ω to 138 Ω with pulse 1, manifesting a Raman spectrum of crystalline GeTe [34]. Subsequently, applying a voltage pulse of 20 V/200 ns (pulse 2) returns the resistance to a high state. These Raman spectra results indicate the successful switching of GeTe between crystalline and amorphous states. The Raman spectra technique can be used to analyze thermal damage [35], as well as the strain [36] and oxidation around the film [37,38], especially in the process of rapid heating and multiple cycles [39]. The thermal distribution of the device, thermal stresses, and oxidation of the heater need to be paid attention to when carrying out characterization. Although this paper is not fully dedicated to these effects, work in the future will focus on these aspects to enhance the performance of the device.

Cycling tests were conducted on the W microheater and GeTe-based phase-change switch based on pulse 1 and pulse 2, as depicted in Figure 11d,e. It can be seen that both the heater and the switch cycled 500 times without failure. The voltage pulse that is required to turn on the switch is 11 V/3 μs, causing the resistance of the device to transition from M Ω to 138 Ω. Subsequently, applying a voltage pulse of 20 V/200 ns returns the resistance to a high state, effectively turning the switch off. The optimized switch was successfully actuated both on and off without any damage to the device, signifying that the heat generated by the microheater elevated the GeTe temperature beyond 720 °C in 200 ns. In contrast, the switch shown in Figure 1 failed to achieve the off state due to stress-induced damage to the device. Figure 8f illustrates the insertion loss and isolation of the device. It can be observed that at 20 GHz, the insertion loss is 1.0 dB and the isolation is 22 dB. As this paper primarily focuses on the optimization of the W heater to actuate the phase-change switch, further research is required to comprehensively optimize the switch’s performance, including achieving lower insertion loss and improved endurance, etc.

Table 4 summarizes the initial resistance R_0_, TCR at 350 °C, and crystalline structure of the four samples. Sample D stands out obviously among these samples due to its positive TCR, lower resistance, and pure α-W phase. Table 5 provides a comparison of the TCR and resistivity of the W film that was applied in this study and other references related to indirectly heated RFPCSs. Limited by the maximum temperature of the sputtering equipment, the TCR and resistivity observed in this study can be further optimized at a higher temperature to enhance the performance of the W film used in phase-change switches compared to other research. Although the preparation process and crystalline structure of W films are not explicitly mentioned in other works on RFPCSs, it can be assumed that the low resistivity and positive TCR suggest that the crystalline phase of W thin films is likely the pure α-W phase, especially at higher deposition temperatures, as indicated in Table 5.

## 4. Conclusions

The role of the W film as a crucial microheater in phase-change switch preparation is explored in this study. The deposition conditions for W films are investigated, leading to the preparation of four samples. Our results show that the substrate temperature and sputtering pressure influence the temperature coefficient of resistance (TCR) of tungsten films. Specifically, films deposited at 650 °C and 1 Pa exhibit a positive TCR with a linear resistance–temperature relationship, while those deposited at room temperature and 0.3 Pa display a negative TCR with a linear relationship. Films deposited at RT or 0.3 Pa show a nonlinear resistance–temperature relationship. The TCR at 350 °C for the four samples is calculated as −1.3, −0.5, −3.4, and +1.2 ppt/°C, respectively. XRD characterization confirms an α-W phase for films deposited at 650 °C and 1 Pa, corresponding to lower resistance due to α-W. The SEM analysis reveals that films deposited at room temperature or low pressure exhibit more stress and larger grains in the α-W phase, leading to lower resistance.

The optimized W microheater, fabricated using modified sputtering parameters and lithography technology, underwent testing without film rupture or device damage at the voltage pulse of 25 V/200 ns. FEM simulations demonstrate that a 25 V/200 ns pulse can achieve a device temperature beyond the melting point of GeTe, thereby fulfilling the conditions for the GeTe phase change to switch off. This is also confirmed by the realization of the off state in an optimized switch measurement. However, the optimized microheater was able to withstand 37 V/200 ns pulses without damage, demonstrating at least a 48% increase in maximum voltage.

Three-dimensional surface profiler inspections reveal numerous spikes and bulges, especially at the edges of the microheater, induced by a 25 V/200 ns pulse causing rapid heating in the central region and a gradual rise in edge temperature. Despite these challenges, the optimized microheater successfully operates the phase-change switch 500 times without damage, requiring a 20 V/200 ns pulse to turn the switch off with MΩ and 11 V/3 μs pulse to turn it on with 138 Ω. The TEM results show that no oxidation or degradation occurs during the operation of the device. The optimized device exhibits insertion loss and isolation of 1.0 dB and 22 dB at 20 GHz. As this study focuses on W heater optimization, further research is needed for comprehensive switch performance optimization.

## Figures and Tables

**Table 1:** Sputtering parameters of tungsten films.

Sample	Sputtering Power (W)	Pressure (Pa)	Temperature (°C)	Ar Flow (sccm)
A (applied in Figure 1)	200	0.3	RT	35
B	200	0.3	650	35
C	200	1	RT	35
D	200	1	650	35

**Table 2:** Calculation results of grain size D and strain ε of four samples.

Sample	Peak 2θ (°)	FWHM β (°)	Grain Size D (nm)	Strain ε (×10^−3^)
A	35.765	0.857	9.7373	11.593
	40.277	1.317	6.4242	15.678
B	35.689	0.787	10.7490	9.359
	40.307	0.439	19.0087	5.950
C	35.768	0.802	10.3999	10.853
	40.293	1.009	8.3864	12.000
D	35.604	0.281	27.2982	3.824
	40.136	0.309	29.6458	3.700

**Table 3:** Related parameters in Comsol simulation.

Material	Coefficient of Thermal Expansion (1/K)	Heat Capacity at Constant Pressure (J/(kg·K))	Density (kg/m³)	Thermal Conductivity (W/(m·K))
W	4.5 × 10^−6^	132	19,350	174
Au	14.2 × 10^−6^	129	19,300	317
Si_3_N_4_	2.3 × 10^−6^	700	3100	170
SiO_2_	0.5 × 10^−6^	730	2200	1.4
Si	2.6 × 10^−6^	678	2320	34

**Table 4:** R_0_, TCR, and crystalline structure of the four samples.

Sample	Slope or First-Order Derivative of Fitted Equation	R_0_ (Ω)	TCR (ppt/°C) at 350 °C	α or β Phase
A (applied in Figure 1)	−0.61	450	−1.3	β + α
B	0.052−0.000246 (T − T_0_)	53	−0.5	β + α
C	0.346−0.00266 (T − T_0_)	155	−3.4	β + α
D	+0.0078	6.6	+1.2	α

**Table 5:** Comparison of TCR and resistivity of W film.

Reference	Deposition Temperature (°C)	TCR (ppt/°C)	Resistivity (nΩ·m)
This work	650	+1.2	400
[20]	N/A	+2.2	143 ± 1
[21]	850	N/A	168
[22]	400 850	+1.3 +3.3	+230 +75.2
[23]	N/A	+1.3	N/A

N/A means not any related information.

## Data Availability

Data will be made available on request.
